# Assessing Top-Down and Bottom-Up Contributions to Auditory Stream Segregation and Integration With Polyphonic Music

**DOI:** 10.3389/fnins.2018.00121

**Published:** 2018-03-07

**Authors:** Niels R. Disbergen, Giancarlo Valente, Elia Formisano, Robert J. Zatorre

**Affiliations:** ^1^Department of Cognitive Neuroscience, Maastricht University, Maastricht, Netherlands; ^2^Maastricht Brain Imaging Center (MBIC), Maastricht, Netherlands; ^3^Cognitive Neuroscience Unit, Montreal Neurological Institute, McGill University, Montreal, QC, Canada; ^4^International Laboratory for Brain Music and Sound Research (BRAMS), Montreal, QC, Canada

**Keywords:** auditory scene analysis, auditory stream segregation, auditory stream integration, polyphonic music, attention, timbre

## Abstract

Polyphonic music listening well exemplifies processes typically involved in daily auditory scene analysis situations, relying on an interactive interplay between bottom-up and top-down processes. Most studies investigating scene analysis have used elementary auditory scenes, however real-world scene analysis is far more complex. In particular, music, contrary to most other natural auditory scenes, can be perceived by either integrating or, under attentive control, segregating sound streams, often carried by different instruments. One of the prominent bottom-up cues contributing to multi-instrument music perception is their timbre difference. In this work, we introduce and validate a novel paradigm designed to investigate, within naturalistic musical auditory scenes, attentive modulation as well as its interaction with bottom-up processes. Two psychophysical experiments are described, employing custom-composed two-voice polyphonic music pieces within a framework implementing a behavioral performance metric to validate listener instructions requiring either integration or segregation of scene elements. In Experiment 1, the listeners' locus of attention was switched between individual instruments or the aggregate (i.e., both instruments together), via a task requiring the detection of temporal modulations (i.e., triplets) incorporated within or across instruments. Subjects responded post-stimulus whether triplets were present in the to-be-attended instrument(s). Experiment 2 introduced the bottom-up manipulation by adding a three-level morphing of instrument timbre distance to the attentional framework. The task was designed to be used within neuroimaging paradigms; Experiment 2 was additionally validated behaviorally in the functional Magnetic Resonance Imaging (fMRI) environment. Experiment 1 subjects (*N* = 29, non-musicians) completed the task at high levels of accuracy, showing no group differences between any experimental conditions. Nineteen listeners also participated in Experiment 2, showing a main effect of instrument timbre distance, even though within attention-condition timbre-distance contrasts did not demonstrate any timbre effect. Correlation of overall scores with morph-distance effects, computed by subtracting the largest from the smallest timbre distance scores, showed an influence of general task difficulty on the timbre distance effect. Comparison of laboratory and fMRI data showed scanner noise had no adverse effect on task performance. These Experimental paradigms enable to study both bottom-up and top-down contributions to auditory stream segregation and integration within psychophysical and neuroimaging experiments.

## Introduction

Listening to an orchestral performance demonstrates the auditory system's extraordinary capability to both segregate and integrate sound sources within a complex mixture of simultaneously playing instruments and background sounds. While most listeners can segregate individual melodic lines, for example a flute and a harp from the mixture, the same excerpt could be differentially perceived by integrating multiple instruments into a single percept, focusing on, for example, their harmonic relationships. Mechanisms contributing to resolving such Auditory Scene Analysis (ASA) challenges have been extensively studied psychophysically and comprehensively described in Bregman's (1990) work, proposing a framework for the perceptual organization of sounds. Stream segregation is responsible for parceling an auditory scene with multiple sound sources into individual acoustic events or auditory streams (McAdams and Bregman, 1979; Bregman, 1990; Micheyl et al., 2007; Ciocca, 2008). Segregation and integration of sources within mixtures of spectrally and temporally overlapping sounds is mainly driven by physical (i.e., bottom-up) differences, and may be further facilitated by, among others, selective attention (i.e., top-down modulations; Bregman, 1990; Brochard et al., 1999; Shamma and Micheyl, 2010). In polyphonic music, pitch and instrument timbre differences have been indicated as prominent examples of bottom-up cues (for example, Bregman and Pinker, 1978; Wessel, 1979; Cusack and Roberts, 2000; Deutsch, 2013; Marozeau et al., 2013; McAdams, 2013a,b), with top-down attention potentially modulating sound feature representation(s) or general source salience (Carlyon, 2003; Cusack et al., 2004; Carlyon and Cusack, 2005; Sussman et al., 2007; Besle et al., 2011; Lakatos et al., 2013; Riecke et al., 2016).

Polyphonic music very well exemplifies ASA in naturalistic complex auditory scenes as encountered by many listeners on a daily basis, comprising multiple sources from various instruments combined with changing degrees of spectral-temporal overlap. However, contrary to traditional cocktail-party designs, polyphonic music stimuli not only permit studying classical source segregation, they also add the possibility to investigate the relatively neglected ASA aspect of stream integration across (complex) sounds (Sussman, 2005; Deutsch, 2013; Uhlig et al., 2013; Ragert et al., 2014). Even though initial segregation of music voices is probably necessary to perceive polyphony, the simultaneous percepts of coherent melodic lines is most likely achieved by integration (Bregman, 1990; Gregory, 1990; Bigand et al., 2000), which is potentially modulated by top-down influences. A general performance benefit is observed on divided attention tasks employing polyphonic music, as compared to many experiments using other types of stimuli such as multiple simultaneous speech streams (for example, Bigand et al., 2000). Observed superior performance is hypothesized to be driven by the existence of both a perceptual and structural relationship between the multiple music voices comprising counterpoint/polyphonic pieces. Counterpoint music contains structural relationships both across the notes of individual voices (i.e., horizontal coherence) and between the individual melodic lines (i.e., vertical integration). Music voices, therefore, need to have sufficient commonalities to express their musical relationship and allow their integration, by, for example, top-down processes, even though the need remains for preserving ample differentiating factors, such as pitch or timbre, to allow for their segregation.

The majority of psychophysical and neuroscientific studies on ASA have been implemented using relatively elementary auditory scenes with, for example, tones in noise or multiple alternating tone sequences (for reviews see, Bregman, 1990, 2015; Carlyon, 2003; Ciocca, 2008; Alain and Bernstein, 2015). While music listening and processing has been studied extensively in recent years (for reviews see, Peretz and Zatorre, 2005; McDermott and Oxenham, 2008; Zatorre and Zarate, 2012), very few have attempted to investigate ASA employing more complex and realistic polyphonic music (Janata et al., 2002; Ragert et al., 2014). Conversely, some studies did apply ASA segregation mechanisms to explain polyphonic/multi-part music perception (for example, Deutsch, 2010, 2013), even though no tasks have been developed to allow the study of ASA with naturalistic stimuli. The present work tries to address these factors and describes two psychophysical experiments employing polyphonic music, aimed at introducing a more ecologically valid stream segregation and integration paradigm as compared to the commonly used schematic streaming designs (for example, Bregman, 1990, 2015; Carlyon, 2003; Ciocca, 2008). We introduce a task for investigation of both stream integration and segregation with custom-composed polyphonic music stimuli, and, contrary to most previous ASA studies, provide a selective attention behavioral performance metric for both the segregation and integration of scene elements, allowing behavioral validation of task performance. The polyphonic music used was specifically designed to remain as close as possible to highly controlled stimuli typically employed in more schematic stream segregation tasks, while still being perceived as a complex music stimulus frequently encountered by listeners. Designing stimuli in this way aids task interpretation and integration within existing ASA literature, while the use of full complex music stimuli, for example extracted from existing compositions, would render the literature integration more difficult. In the current study we will introduce the task, demonstrate its validity, including its use with non-musically trained subjects, document its reliability, and make both task and stimuli available to the community for future use.

When ample physical differences between sound sources exist, such as in the case of instruments with different timbres, the integrative condition is not expected to show reduced performance compared to the segregative conditions (for example, van Noorden, 1977; Bregman, 1990, 2015; Moore and Gockel, 2002). To test this hypothesis, in Experiment 1 the participant's locus of attention was varied via visual instructions. While listening to polyphonic music, participants were asked to attend individual instruments or the aggregate (i.e., both instruments) and detected rhythmic modulations incorporated within or across instruments. Our main goal was to develop a task that was challenging to non-musicians while equating difficulty across conditions, maintaining high correct scores and preserving the possibility to monitor participant's locus of attention. By reducing instrument timbre differences, more challenging segregation conditions can be created (for example, van Noorden, 1977; Melara and Marks, 1990; Gregory, 1994; Cusack and Roberts, 2000; Moore and Gockel, 2002; Bey and McAdams, 2003; Sussman, 2005), possibly improving performance on the less demanding task of source integration. Conversely, an increase of timbre difference could facilitate the source segregation and decrease integration performance, giving rise to an instrument-timbre interaction: more attentional resources are recruited for segregating sources with smaller physical (i.e., bottom-up) differences compared to their integration, while less attentional resources are required for segregation than integration when there are larger physical differences. Such possible effects are tested in Experiment 2, which adds a bottom-up manipulation to the attentive modulation framework of Experiment 1 by introducing a three-level change in instrument timbre distance.

Both experiments were specifically designed to facilitate eventual investigation of top-down and bottom-up contributions to stream segregation and integration processes for complex sounds using both behavioral and neuroimaging paradigms. To generalize the task to the latter application, Experiment 2 was additionally validated within the functional Magnetic Resonance Imaging (fMRI) scanner environment. Subjects were tested either in a sound attenuated chamber without background scanning sequence noise (LAB group), or during a multi-session fMRI experiment (SCAN group) with a continuous imaging sequence, allowing assessment of whether group-level task performance was affected by the addition of scanner noise to the auditory scene. The factor of background noise in fMRI scanning (Belin et al., 1999; Amaro et al., 2002; Hall et al., 2014; Andoh et al., 2017) is often ignored, but becomes of special significance in the case of stream segregation studies.

## Methods

### Stimuli

Twenty polyphonic counterpoint music pieces were custom-composed in close collaboration with a composer, providing desired control over acoustical content while remaining recognizable as polyphonic music. All pieces were 28 s long and included two voices written in treble and bass clef, respectively synthesized in bassoon and cello, at tempo 60. Compositions were controlled for, among others, pitch distance between voices (never touching or crossing), rhythmic modulations, and pitch modulation size (Supplementary Figure 1). Tempo of 60 beats per minute was selected among other faster alternatives to allow not musically-trained individuals to detect temporal modulations within the music. Sixty-two additional unique pieces were custom-written for training and testing purposes, of which six were composed meeting the exact same requirements as the experimental pieces. All training music was unrelated to the experimental compositions and not repeated anywhere other than in their respective training round or the pre-test (see Participant Selection and Training). Music pieces were synthesized from Musical Instrument Digital Interface (MIDI) files in mono for bassoon (treble clef) and cello (bass clef) independently, sampled at 44.1 kilo Hertz with a 16Bits resolution using Logic Pro 9 (Apple Inc., Cupertino, California, USA). Stimuli were combined into polyphonic pieces, Root Mean Square (RMS) equalized, and onsets-offsets exponentially ramped with 100 ms rise-fall times. Stimulus processing and manipulation after sampling was performed with custom-developed MATLAB (The MathWorks Inc., Natick, Massachusetts, USA) codes.

**Figure 1:**
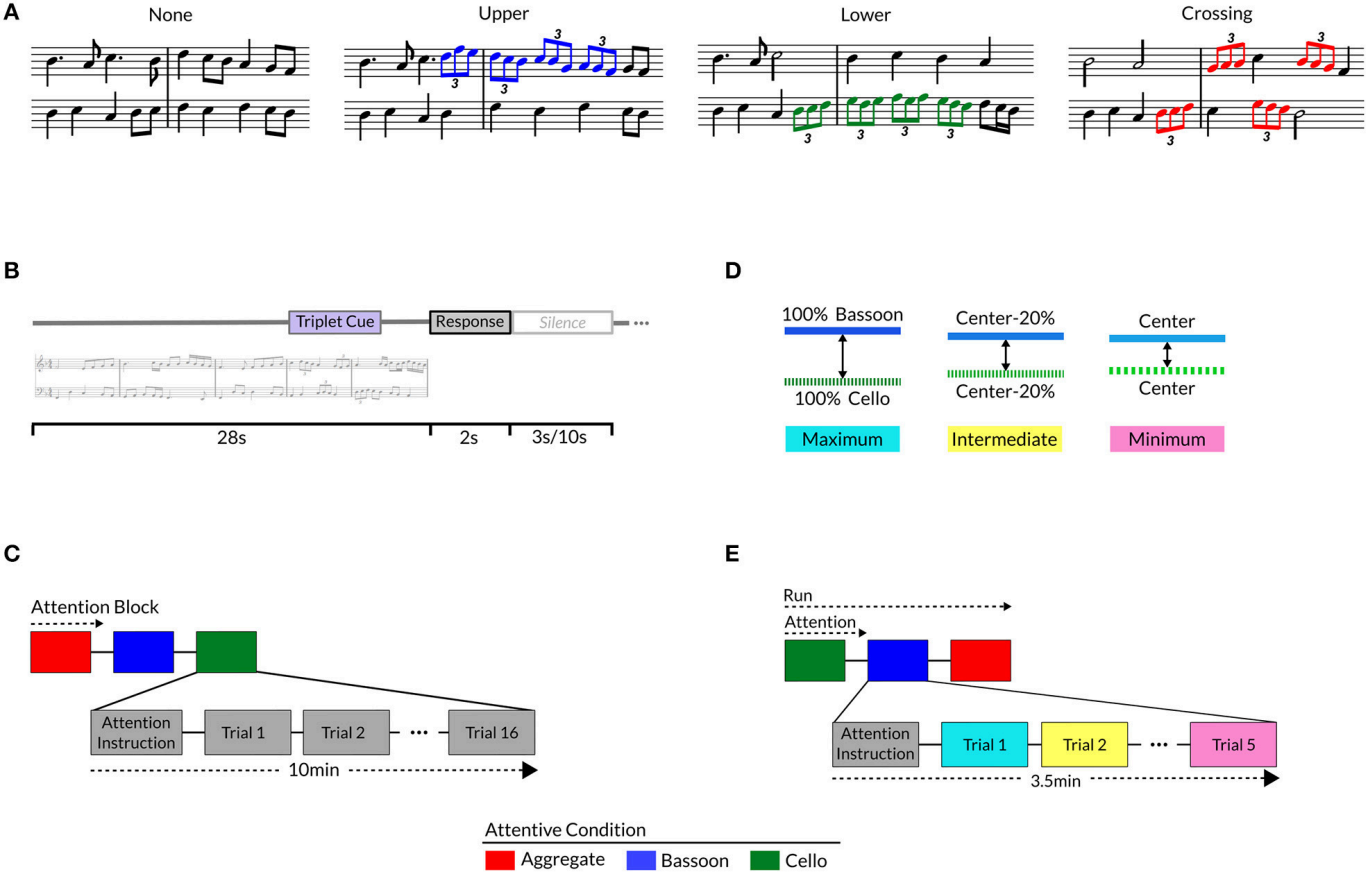
Different Triplet versions for each music composition **(A)**: no triplets, upper voice (i.e., bassoon; blue notes), lower voice (i.e., cello; green notes), crossing voices (red notes). Trial buildup **(B)** with stimulus, response window, a 3 s (Experiment 1 and Experiment 2 LAB) or 10 s (Experiment 2 SCAN) silence; Experiment 1 **(C)** and 2 **(E)** trials presented in attentive blocks, preceded by attention instruction and silence. Experiment 2 instrument timbre manipulation **(D)** per triplet version: original timbre (maximum; blue), morph perception center-point (minimum; pink), or morphed 20% more toward maximum from minimum (intermediate; yellow).

To provide a control on the locus of selective attention, participants detected rhythmic modulations comprising a pattern of triplets incorporated in the polyphonic music. Triplets, in our specific case, are defined as three eighth notes played in the time of one beat, typically perceived by (non-musically trained) listeners as a “speeding-up” of the music compared to its flanking notes. Temporal modulations in the form of triplets were chosen because they are orthogonal to pitch changes, facilitating detection by non-musically trained listeners and providing maximum independence from pitch-based segregation mechanisms. Patterns detected by subjects consisted of four eighth-note triplets in a row, comprising a total duration of 4 s, and were either present within bassoon (Figure 1A, blue notes), or cello (Figure 1A, green notes), or occurred across voices (Figure 1A, red notes), or were not present. When patterns crossed voices, they started randomly with the first triplet in bassoon or cello and accordingly alternated between voices; when located within a single voice, all triplets were only present inside the respective instrument's melody. Patterns were pseudo-randomly incorporated in the second half of each excerpt between 14 and 19 s, and surrounding music contained no specific information concerning pattern location or presence. To prevent triplets standing out too obviously from neighboring notes, the patterns followed the excerpt's melody. Adopting such a design resulted in stimuli which only differed as to the inclusion and position of triplets. Incorporating the same four-triplet pattern within and across voices ensured participants detected the same pattern independently of condition. Experimental stimuli are available for download via the Zatorre lab's website^1^.

Instrument timbre was manipulated for each melody separately via interpolation using the STRAIGHT^2^ vocoder speech manipulation software tool in MATLAB (Kawahara and Matsui, 2003). Morphing was performed individually for each melody (i.e., voice), interpolating the melody from the version played by a bassoon toward that played by the cello, hence modulating the melody's timbre/instrument. To achieve this, time-frequency landmarks were created on each instrument's synthesized melody, with landmark time centered on the middle of each note and frequency tagged as the note's f0. Within each music voice, instrument timbres were morphed by logarithmic interpolation of spectral density and aperiodicity. For a subset of five Experiment 1 compositions (1, 4, 5, 8, and 10), instrument timbre was manipulated by morphing individual voices played by their original instrument toward the other instrument in 10% increments, for example leading to the following combinations: 100% bassoon and 0% cello, 90% bassoon and 10% cello, 80% bassoon and 20% cello, *et cetera*. Resulting stimuli were combined into polyphonic pieces, RMS equalized, exponentially ramped with 100 ms rise-fall times, and filtered per individual channel with Sensimetrics equalization filters in MATLAB.

### Participants

Twenty-nine adult volunteers (18 women; age 23.4 +/− 3.7 years, *mean* +/− *standard deviation*) with self-reported normal hearing, motor, and vision abilities participated in Experiment 1. None of the participants spoke a tonal language and all had less than 2 years of (formal) music training on a lifetime basis with instruments other than bassoon or cello, as assessed via the Montreal Music History Questionnaire (Coffey et al., 2011). After completing Experiment 1, a group of 19 listeners also participated in Experiment 2 (13 females, age 22.7 +/− 2.6 years). Participants in Experiment 2 were initially recruited for a fMRI experiment, however, when not eligible for MRI due to safety concerns, the experiment was completed in a sound-attenuated chamber (*N* = 9; LAB). The remaining subjects (*N* = 10; SCAN) performed the task inside the fMRI scanner over the course of two or three scanning-sessions. Subject assignment to LAB or SCAN group was solely depended on their MRI eligibility, and hence should not have resulted in any bias with respect to task performance ability. Volunteers were mostly students recruited from McGill University (*N* = 7) or Maastricht University (*N* = 22), with both McGill and Maastricht volunteers participating in Experiment 1, and a subgroup of Maastricht volunteers in Experiment 2. Subjects provided written informed consent and experimental procedures were approved by the ethical committees of each university.

### Experiment 1

#### Task

Participants completed a forced-choice delayed-response target detection task within or across music voices, attending to the same instrument(s) during a block of 16 trials (Figure 1C); before initiation of each block they were visually instructed to attend to the bassoon, cello, or aggregate. After each stimulus ended, listeners indicated via a button press whether the triplet pattern was present in the instrument(s) instructed to be attended or not. Post-stimulus responses were adopted to reduce the influence of cognitive decision and motor processes in the stimulus presentation window, specifically beneficial for task employment in neuroimaging experiments. Depending on the attentive condition, triplet presence and/or position differed: in the attend to both instruments condition, half of the trials had triplets crossing over the voices and half contained no triplets; in the attend to bassoon condition, half the trials contained a target pattern in the bassoon voice, as a control one-fourth of the trials contained triplets in the unattended (cello) voice, and one-fourth of the trials contained no triplets; similarly, in the attend cello condition half the trials comprised triplets in cello, one-fourth in bassoon and one-fourth no triplets. Equal distribution between target and no-target trials was adopted to prevent response bias. No-triplet trials along with triplet patterns in the opposite voice were employed to check for false alarms and whether subjects switched attention between instruments as opposed to exclusively focusing on a single voice. Furthermore, opposite-voice patterns provided information if the correct instrument was attended and, in case of a large number of misses, allowed determining whether triplet detection in the opposite voice was achieved. Even though it is not possible to determine with absolute certainty whether a subject was attending to the cued instrument(s) in the various conditions, high performance on the task in conjunction with the above-mentioned measures does provide a strong indicator of a subject's capacity to segregate and integrate music streams.

Trial duration was 33 s and comprised a stimulus of 28 s, response window of 2 s, and a 3 s silence (Figure 1B). After nine trials simulating the experiment, 96 trials from 16 (*N* = 7 participants; McGill University volunteers), or a sub-set of 10 (*N* = 22; Maastricht University volunteers), unique compositions were presented over six attentive blocks. Each stimulus block represented a condition and all conditions were repeated twice, each time with a unique stimulus order. Within a participant the same conditions could not follow each other and the order within the first and second block of three conditions had to be unique (e.g., ACB-CAB); condition ordering was balanced across subjects. Stimulus presentation order was pseudo-random, controlling that within a block of 16 trials, stimuli of the same composition could not follow one another, and a composition could not be repeated more than once. Stimuli were delivered through Shure SRH1440 professional open-back headphones (impedance 37Ω; Shure Inc., Niles, Illinois, USA) at approximately 85 dB SPL in a sound-attenuated chamber via a Creative Sound Blaster Audigy 2ZS (Creative Technology Ltd., Singapore) sound card, employing Presentation 17.0 (Neurobehavioral Systems Inc., Albany, California, USA) for stimulus presentation and response recording. Before participation in the experiment, subjects completed a training session and a pre-test for learning assessment; see Participant selection and Training for details.

### Experiment 2

#### Task

In Experiment 2, a manipulation of bottom-up information (timbre) was added to the Experiment 1 design, keeping all task and stimulus aspects not otherwise mentioned below equal to Experiment 1. The difference between instrument timbres was varied across three discrete levels while subjects performed the attentive task as described in Experiment 1. Timbre morphs were combined to create three instrument timbre distances: each melody played by their respective original instruments (i.e., no timbre manipulation; maximum; Figure 1D, blue), minimum timbre difference between instruments (minimum; Figure 1D, pink), and 20% closer toward maximum from the minimum distance values (intermediate; Figure 1D, yellow). Minimum timbre distance between voices was determined perceptually for each subject in a separate experiment, rating their instrument perception for all timbre morphs per voice (see Timbre Perception). Individual matching of timbre distance was adopted to account for subject variation with respect to their perceptual center points, based on a pilot experiment suggesting such differences.

Participants attended the same instrument(s) during an attentive block of five trials, each comprising a stimulus of 28 s, response window of 2 s, and a 3 s (LAB) or a 10 s (SCAN) post-silence (Figure 1B). After several practice trials, a total of 90 (LAB) or 135 (SCAN) trials were presented across six (LAB) or nine (SCAN) runs of three attention blocks each (Figure 1E). Composition version distribution across conditions was equal to Experiment 1, with the only exception that eight target trials and seven control trials were included in a run. Within the bassoon and cello conditions, this distribution resulted in control trials comprising uneven numbers of no-triplet versions and opposite-to-attention-voice triplet versions (three or four of each). To mitigate any effects of such imbalance, their numbers alternated across experiment repetitions: three-four or four-three. Stimulus order was pseudo-random, controlling that each timbre distance version of each composition was covered by all conditions over the course of three runs/nine attentive blocks. Within attention blocks, compositions could occur only once, same timbre distances could not follow, a composition's timbre distance had to occur at least once, and the same timbre distance could not occur more than twice. Within a run, stimuli could only occur once, the first stimulus of a consecutive attentive block could not be the same as last of the previous, and number of timbre distance occurrences for each composition had to be equal. In one experiment repetition (i.e., three runs/nine attentive blocks), all conditions uniquely occurred at each position within the three-block sequence, for example: ABC-BCA-CAB. For all three experiment repetitions (i.e., nine runs/27 attentive blocks), condition order blocks appeared in all positions, for example: repetition (1) ABC-BCA-CAB, (2) BCA-CAB-ABC, and (3) CAB-ABC-BCA. Across participants, condition run order was balanced, for example: participant (1) BCA-CAB-ABC vs. (2) CBA-BAC-ACB. Stimuli were presented through Sensimetrics (Sensimetrics Corporation, Malden, Massachusetts, USA) S14 ear-buds at approximately 83 dB SPL via a Creative Sound Blaster Audigy 2ZS sound card (LAB). During the fMRI sessions (SCAN) stimuli were presented via Sensimetrics S14 ear-buds and a Creative Sound Blaster X-Fi Xtreme Audio sound card at around 94 dB SPL, a gain of approximately 30dB over the scanner sequence noise.

#### Timbre perception

During a separate session, each participant's minimum instrument timbre distance point was perceptually determined by rating their perception of all timbre morphing steps per individual voice. Each subject was presented with all 10% morph steps to allow assessment of their changing percept, as we were not aware of any other data quantifying these timbre modification effects. Listeners were first habituated to the unaltered instrument timbre, corresponding to maximum timbre difference across voices, with 10 stimuli per instrument. Next, we assessed with 10 different test stimuli per instrument whether they correctly identified the timbre of both bassoon and cello. Subjects ranked their timbre morph perception of 176 trials on a one-to-five scale: 1 = bassoon, 3 = intermediate, 5 = cello. Morphs were pseudo-randomly presented over eight equal-length blocks, controlling that the same composition or the same voice did not follow each other. The rating scale was visually presented throughout the experiment, fading to the background during stimulus presentation and turning brighter for the 2-s response window which was followed by a 2-s silence. Stimuli spanned all 10% timbre morphing steps, giving a total of 11 versions per instrument voice per composition. Perceptual midpoints were determined by fitting a sigmoid to all voice's data points *r*, with minimum *a* = 1 (bassoon percept rating) and maximum *b* = 5 (cello perception rating). Center point *x*50 as well as slope *m* was estimated per individual voice by non-linear regression with iterative least squares estimation and initial values *m* = 1 and *x*50 = voice mean rating:

$$S=1+\frac{b-a}{1+10^{(x50-r)*m}}$$

Sigmoid center point *x*50 was subsequently selected as the voice's timbre perception center, and morphs were combined into the three instrument timbre distances (see Experiment 2 Task): maximum (Figure 1D, blue, i.e., original instruments), minimum (Figure 1D, pink, i.e., perceptual center for each instrument), and intermediate (Figure 1D, yellow, i.e., perceptual center minus 20 percent).

### Participant selection and training

Participants received Experiment 1-specific training, exposing them over 10 training-rounds to music of increasing complexity, ranging from scales including isolated triplets in a single voice until polyphonic melodies at melodic complexity equal to experimental stimuli and including the triplet pattern (Table 1). Training rounds consisted of initial instructions including examples and, to assess learning, several test stimuli with varying performance requirements (see Table 1). Protocols provided the option to repeat examples as desired and were developed to be self-explanatory as well as adaptive to participant performance. Feedback was presented after each trial requiring response, and if training round test performance was insufficient, the full round could be repeated maximum twice. After training, generalization was tested via a pre-test simulating a shortened version of Experiment 1, employing four custom written compositions across 24 trials. Training was completed in two different groups, the first (*N* = 14) started with training and completed Experiment 1 if requirements were met (*N* = 10). The second group (*N* = 68) first completed the perceptual rating experiment of instrument timbres and if perceiving differences between morphs (*N* = 21), continued to the training phase, which was completed successfully by the 19 subjects who participated in Experiment 2. Pooling over both groups, 83 percent of participants could be successfully trained.

**Table 1:** Training rounds and pre-test with their respective triplet types, melody complexity, included instruments, triplet location, task and number of test stimuli, and minimum score needed to pass the round. R = training round, PA = rate triplet(s) as present or absent, Exp = experimental task.

**ID**	**Triplet type**	**Melody**	**Instrument(s)**	**Triplet location**	**Task (test-stim)**	**Min. correct (%)**
R1	Single	Scales	Bassoon	Individual Voice	PA (6)	100
R2			Cello			
R3		Basic	Bassoon			
R4			Cello			
R5		Complex	Bassoon			
R6			Cello			
R7	Pattern		Bassoon & Cello	Bassoon	PA (5)	85
R8				Cello		
R9				Crossing		
R10		Experimental		Variable	PA (8)	
Pre-test					Exp (24)	

### Analysis

Responses to both experiments were classified as hits *H*, misses *M*, false alarms *FA*, and correct rejections *CR*. Due to scores occurring close to ceiling, d-prime values were edge-corrected (see for example, Stanislaw and Todorov, 1999) for visualization, Bayesian model initiation (see below), and comparison purposes only:

$$d_{ec}^{\prime }=\Phi^{-1}(\frac{H+0.5}{H+M+1})-\Phi^{-1}(\frac{FA+0.5}{FA+CR+1})$$

where Φ^−1^ denote the inverse normal cumulative distribution function with μ = 0 and σ = 1.

Differences between experimental conditions were statistically evaluated with an ANOVA-like hierarchical Bayesian model including multiple grouping variables, with each subject contributing measures to all groups. Hierarchical models are particularly well suited to describe data from individuals within groups, comprising parameters for each individual as well as higher-level group distributions, allowing integrating group and individual parameters in the same model. Such an integration has several advantages, among which the possibility to correctly estimate the variance due to subject effects with different/smaller sample sizes across groups. Additionally, these models can seamlessly handle participant performance close to ceiling, an unequal number of trials per condition, and possible heteroscedastic variances across conditions (based on a pilot experiment suggesting such variance differences). One of the strengths of incorporating Bayesian methods in a hierarchical framework, comprises the possibility of reallocating the model's parameter value credibility over more restrictive options when more data is added to the model, providing as output a distribution of credible parameter values (e.g., a condition's effect) which inherently capture the estimated parameter's uncertainty. Furthermore, the use of Gibbs sampling in the Bayesian framework allows to perform inference on those models which cannot be analytically derived, as is the case for our model, preventing the need for approximations/simplifications to make the problem tractable. Estimation and inference with hierarchical models presents many challenges in the standard (frequentist) setting, therefore, in this work, both for computational reasons and model flexibility, we chose to employ instead Bayesian estimation.

The use of a standard frequentist analysis on d-prime would be suboptimal in our case, due to several subjects performing at ceiling. It would be necessary to perform such analysis on edge-corrected d-prime values, since ceiling d-prime is infinite, which, among others, leads to a loss of sensitivity in the higher ranges. Furthermore, submitting an estimated d-prime to a standard statistical test, for example an ANOVA, would rely on the assumption that all measurements have similar uncertainty. However, this is not the case for d-prime values close to ceiling, which have a different variance compared to those in the lower ranges. The most employed statistical tool to handle such heteroscedasticity is a hierarchical (i.e., mixed effects) model, which can weigh different measurements based on their uncertainty and produce a reliable population estimate. These models can be estimated in a frequentist or a Bayesian framework, given the model considered in our work the Bayesian approach was most suited. Bayesian hierarchical models provide further advantages over both frequentist and non-hierarchical models, however full coverage of strengths, weaknesses, and differences between models is beyond the scope of this paper; for an introduction to Bayesian data analysis, see Kruschke (2014).

Hierarchical model parameter estimate ranges will be expressed as Highest Density Intervals (HDIs; for example, Kruschke, 2014), whose range spans x-percent of the parameter estimation distribution and is analogous to the parametric confidence interval; an HDI of, for example, 95% from a standard normal distribution would extend from −1.96 to 1.96. When an effect's HDI range includes zero it indicates there is probably no effect of respective condition. Models were estimated with JAGS^3^ (Just Another Gibbs Sampler, version 3.3.0) via its Matlab integration MATJAGS^4^ (version 1.3.1) employing Gibbs sampling Markov Chain Monte Carlo (MCMC) simulations. JAGS models are defined by nodes, which in the model definition are written as either a stochastic relationship “~” (i.e., random variable), or a deterministic relation “←” where the respective node value is determined by its parents. Each model was estimated with 10.000 MCMC samples and a burn-in of 2.000 samples. Edge-corrected d-primes were used as model initiation variables, providing condition specific values for each subject from which to start model fitting.

Correlation of subject performance between Experiment 1 and Experiment 2 for those subjects who completed both experiments (*N* = 19) was performed using Spearman linear rank correlation on correct rates over all trials for Experiment 1 and maximum morph distance only trials for Experiment 2. Experiment 2 correlation between correct rates for all trials and subject morph effect, was calculated with a Spearman linear rank correlation by subtracting accuracy for all minimum morphing distance trials from all maximum distance trials. Correct rates were selected due to a different number of trials between comparisons, hence a difference in their maximum edge-corrected d-prime values.

### Hierarchical model specification

Experiment 1 model is a simplification of the Experiment 2 model (Supplementary Figure 2), hence only the latter is discussed here in detail. Subject observations per condition were labeled as measurements *m*, three attention and three timbre distance conditions resulted in nine samples per participant and a total of *m*=171. Hits (*H*) and false alarms (*FA*) were, respectively, binomially modeled with the number of hits *n*_*hit*_, trials including triplets *n*_*tri*_, false alarms *n*_*fa*_, and trials excluding triplets *n*_*ntri*_; note that this modeling of d-prime is not equivalent to the edge-correction described above. Hit and *FA* distributions were transformed using the standard normal cumulative distribution function Φ, within which the bias-model (*bias*_*m*_) links to the d-prime model. By modeling *H* and *FA* with the Binomial distribution, we assume all trials are independent and identically distributed:

$$\begin{array}{l} \;H\sim Bin(n_{hit},n_{tri})\\ a_{hit}\leftarrow \Phi (0.5+d_{m}^{\prime }-bias_{m})\\ \;FA\sim Bin(n_{fa},n_{ntri})\\ a_{fa}\leftarrow \Phi (-0.5*d_{m}^{\prime }-bias_{m}) \end{array}$$

Group d-prime values over measurements *m* were modeled as normally distributed with mean $\mu_{d^{\prime },m}$ and variance $\sigma_{d^{\prime },attn}$, by which we assume equal variance across subjects and timbre conditions but not for attentive conditions:

$$\begin{array}{l} d_{m}^{\prime }\sim N\left(\mu_{d^{\prime },m,},\sigma_{d^{\prime },attn}^{2}\right)\\ \sigma_{d^{\prime },attn}\sim Unif(0,4) \end{array}$$

Expected values of the d-prime distribution were estimated with an ANOVA-like model, employing normally distributed parameters: intercept β_0_, attention β_*att*_, timbre β_*timb*_, attention-by-timbre interaction β_a*ti_, and subject β_*subj*_:

$$\begin{array}{l} \mu_{d^{\prime },m}=\beta_{0,m}+\beta_{att,m}*x_{m}+\beta_{timb,m}*x_{m}+\beta_{a*ti,m}*x_{m}\\ \;+\;\beta_{subj,m}*x_{m}\\ \;\beta_{0,m}\sim N(0,1000)\\ \;\beta_{att,m}\sim N(0,\sigma_{\beta_{1}^{2}});\sigma_{\beta_{1}}\sim \Gamma (1.64,0.32)\\ \;\beta_{timb,m}\sim N(0,\sigma_{\beta_{2}^{2}});\sigma_{\beta_{2}}\sim \Gamma (1.64,0.32)\\ \;\beta_{a*ti,m}\sim N(0,\sigma_{\beta_{3}^{2}});\sigma_{\beta_{3}}\sim \Gamma (1.64,0.32)\\ \;\beta_{subj,m}\sim N(0,\sigma_{\beta_{4}^{2}});\sigma_{\beta_{4}}\sim \Gamma (1.64,0.32) \end{array}$$

where Γ(*a, b*) denotes a gamma distribution with shape *a* and rate *b*.

Distribution of β_0,*m*_ was centered on zero with large variance to allow a wide range of intercept values, capturing possible d-prime differences between conditions. Beta prior values for all other predictors are limited in their range via variance σ_β_*n*__. Allowing Beta standard deviations to range freely between close to minimum and maximum probability would result in an unrealistically large standard deviation and range. Such an exaggeration would have too large an influence on our data set with only a moderate number of samples. Based on suggestions by Kruschke (2014, Chapter 21) and considerations concerning number of trials as well as d-prime range, gamma prior values were restricted to Γ(1.64, 0.32), which has mode 2 and standard deviation 4. Experiment biases were modeled equivalently to the described d-prime model, however, for conciseness, only the d-prime model is described in detail, even though the bias is an integral part of the full hierarchical model (see Supplementary Figure 2).

Experiment 1 resulted in three measurements per participant, a total of *m* = 87. Hierarchical model implementation was identical to Experiment 2, with only exception being a parameter reduction on $\mu_{d^{\prime },m}$ to intercept, attention, and subject effects:

$$\mu_{d^{\prime },m}=\beta_{0,m}+\beta_{att,m}*x_{m}+\beta_{subj,m}*x_{m}$$

## Results

### Experiment 1

**Figure 2:**
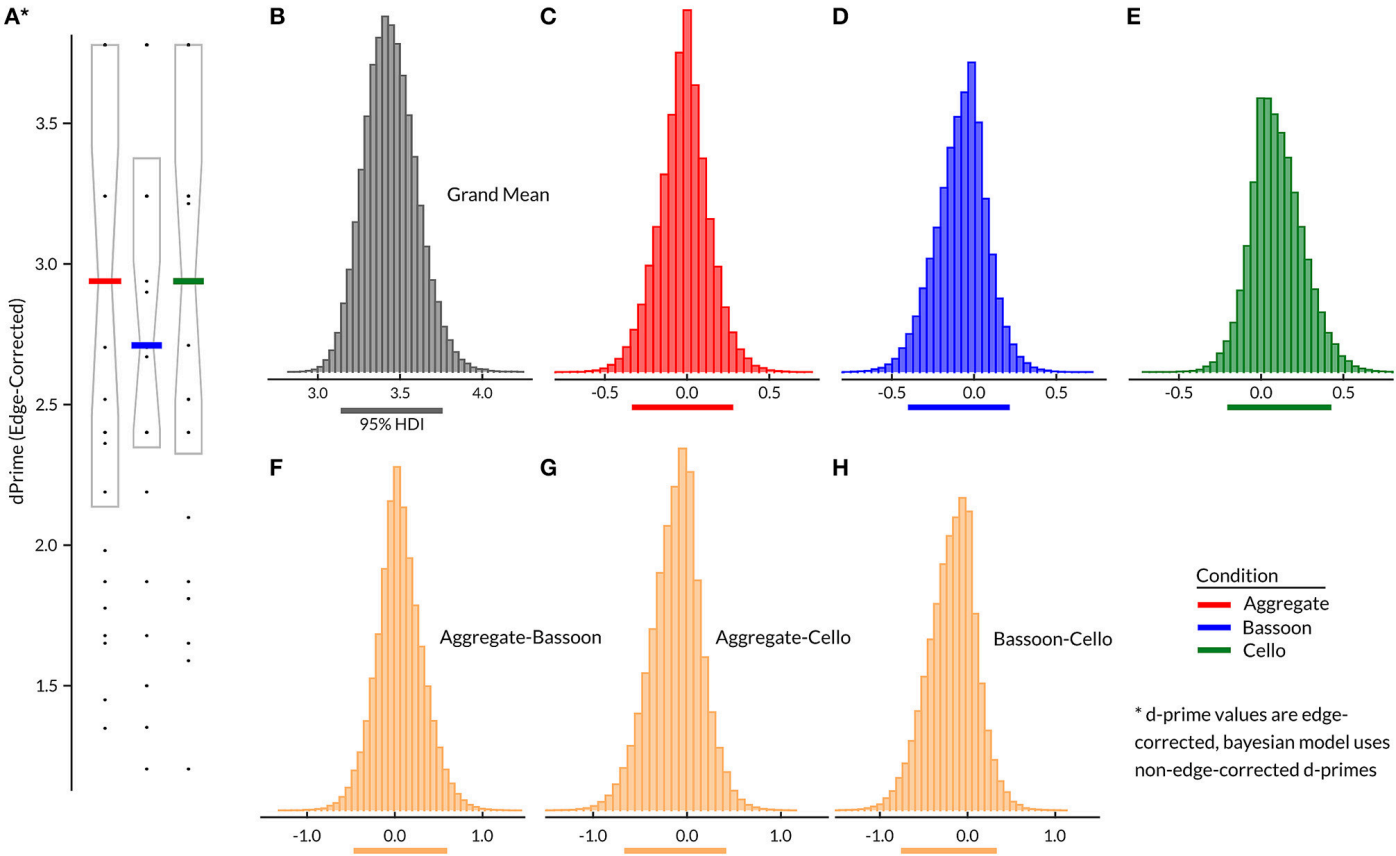
Experiment 1 group results (*N* = 29) for edge corrected d-prime per condition **(A)**, with median (horizontal bar) and 25th–75th percentile. Bayesian hierarchical model **(B–H)**, with group grand mean **(B)**, attention effects **(C–E)**, and contrasts between attention conditions **(F–H)**. Horizontal lines below **(B–H)** indicate 95% HDI.

Inspection of Figure 2A suggests that there was no difference between attention conditions for edge-corrected d-primes in Experiment 1. Bayesian hierarchical model contrasts between all pairs (Figures 2F–H) confirmed that none of the attention effects differed from the grand mean (i.e., subject factor). The grand mean demonstrated that mean performance was relatively comparable across subjects (95% HDI = [3.14 3.76]; Figure 2B); the bias model terms for each condition were strongly centered on zero. Results indicate that the training was successful and subjects were able to complete the task with overall high correct scores, while at the group level task difficulty did not differ across attentive conditions. No systematic group difference was found for False Alarms generated by control trials containing no triplets vs. those with triplets in the unattended instrument. Assessment of accuracy across all trials for included compositions showed comparable values at the group level (Supplementary Figure 1c). Split-half reliability on edge-corrected d-prime values for the full experiment yielded a correlation coefficient of 0.74 (*p* < 0.001).

### Experiment 2

**Figure 3:**
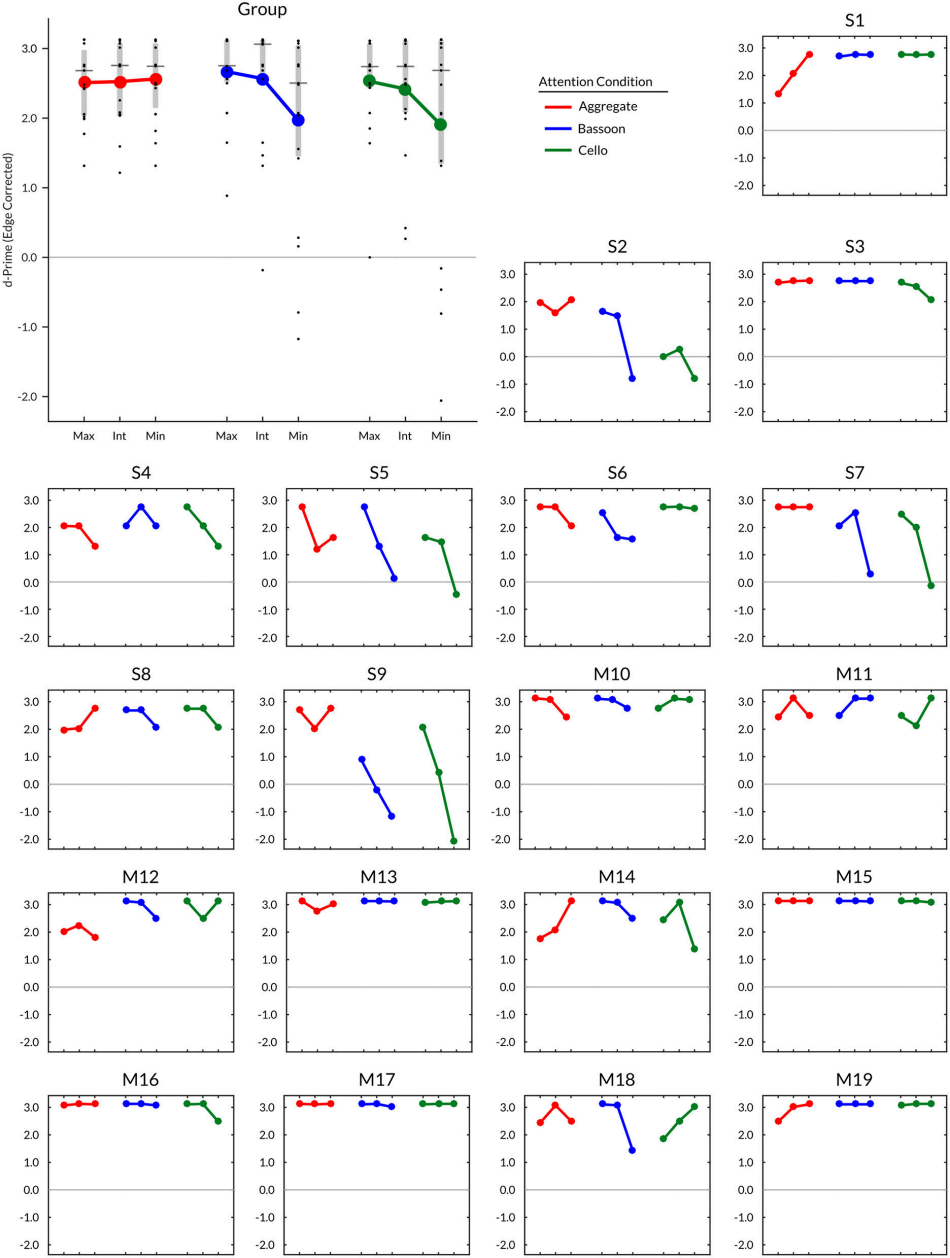
Experiment 2 group and individual edge corrected d-prime results (*N* = 19) per condition and instrument morph distance. Timbre distance: Max, maximum; Int, intermediate; Min, minimum. Vertical gray bars in group plot range 25th–75th percentile; horizontal gray lines median. Participants: S = experiment in sound attenuated chamber (LAB), M = experiment during fMRI (SCAN).

Subject timbre perception center points for the upper voice were determined at 0.4 (i.e., 60% bassoon & 40% cello; *N* = 1), 0.5 (*N* = 11), 0.6 (*N* = 6), and 0.7 (*N* = 1); for the lower voice 0.4 (*N* = 3), 0.5 (*N* = 10), and 0.6 (*N* = 6). Group mean values of edge-corrected d-prime (Figure 3) indicated that intermediate and minimum timbre distance for both bassoon and cello conditions resulted in lower d-prime values compared to their respective maximum timbre distances as well as all three aggregate timbre distances. The observed change in edge-corrected d-prime values was caused by both an increase in False Alarm rate and a decrease in Hit rate (Supplementary Figure 4), confirming effects were not driven by participant bias; no False Alarm bias was found across subjects for trials with no triplets vs. unattended instruments. Accuracy levels across all trials for included compositions were comparable at group level (Supplementary Figure 1d). Edge corrected d-prime scores computed over all trials resulted in a split-half correlation of 0.84 (*p* < 0.001), and subject performance on Experiment 2 was predictable by Experiment 1 scores (*r* = 0.49, *p* = 0.031; **Figure 5A**).

**Figure 4:**
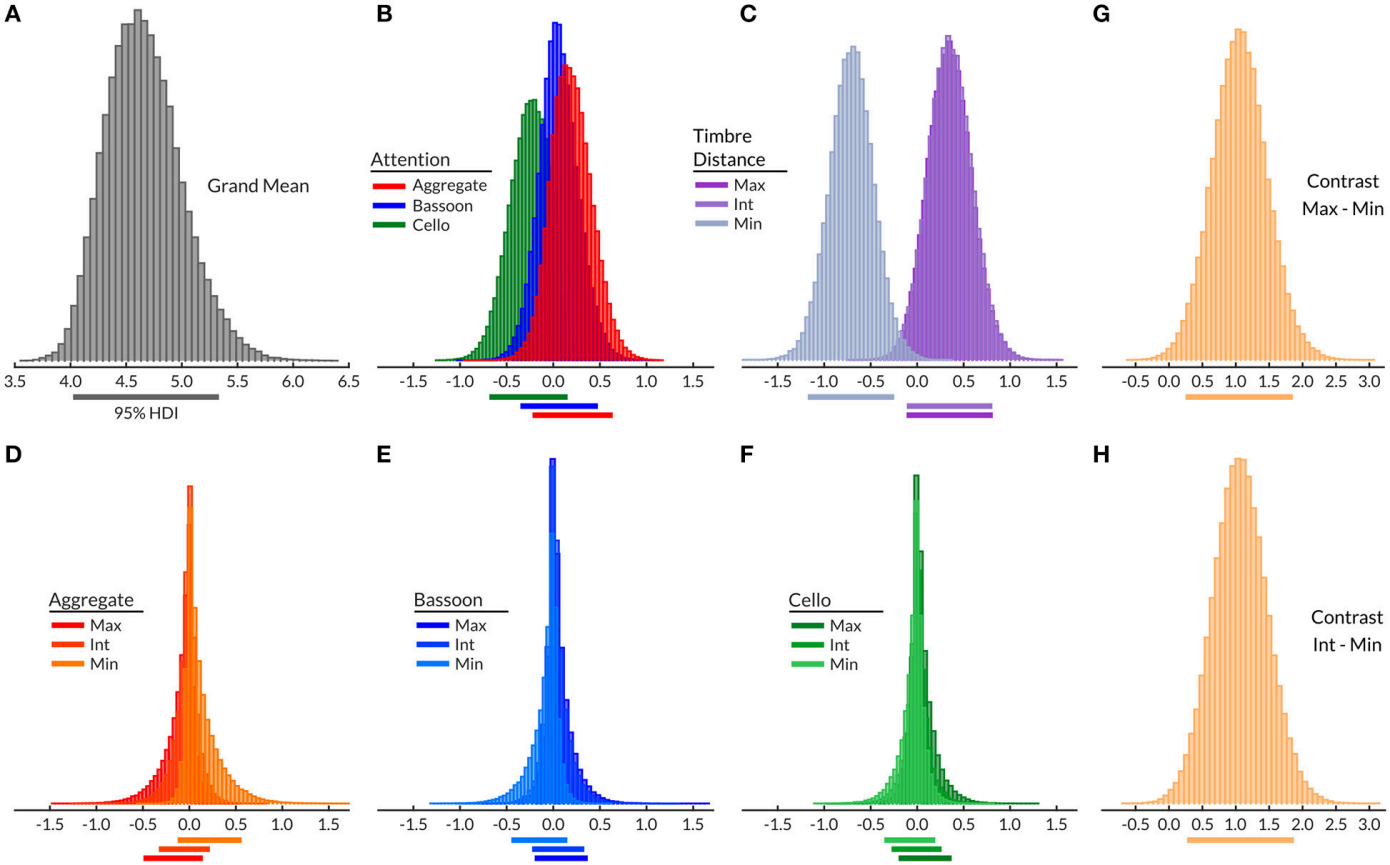
Experiment 2 Bayesian hierarchical model grand mean **(A)**, attention effects **(B)**, morphing effects **(C)**, morphing effects per attentive condition **(D–F)**, all contrasts with a 95% HDI differing from zero (**G,H**; see also Supplementary Figure 3). Timbre distance: Max, maximum; Int, intermediate; Min, minimum. Horizontal lines below histograms, 95% HDI.

Inspection of the Bayesian model grand mean ([4.02 5.34]; Figure 4A), demonstrated a moderate mean performance variation across subjects. Attention main effect did not differ from the grand mean (Figure 4B), nor did subsequent attentive condition contrasts (Supplementary Figure 3) indicate a main effect of attention. Timbre distance effect (Figure 4C) of the minimum timbre difference condition did differ from zero [−1.18 −0.24], indicating that this condition may be more difficult than both the maximum and intermediate timbre distances. Respective contrasts confirmed that the timbre distance main effect was driven by the minimum timbre distance, with both maximum-minimum ([0.25 1.87]; Figure 4G) and minimum-intermediate ([0.27 1.88]; Figure 4H) differing from zero. The within-attention condition timbre distance effects (Figures 4D–F) and their respective contrasts (Supplementary Figure 3), yielded no differences between timbre distance conditions. This observation confirmed that within attention condition timbre distances did not differ in difficulty; the model bias terms were centered on zero and showed no effect of condition.

**Figure 5:**
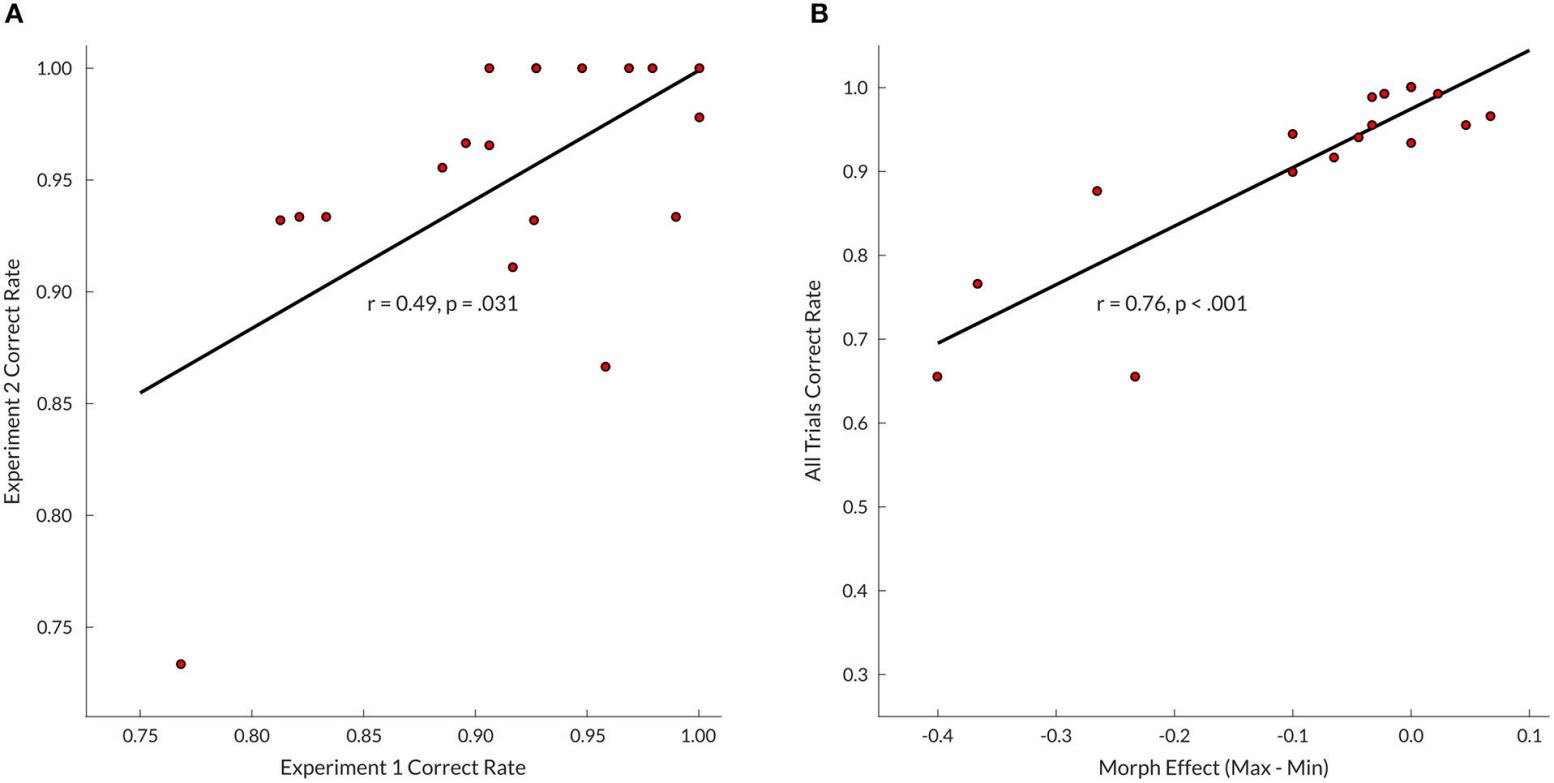
**(A)** Experiments 1 and 2 (maximum timbre distance trials only) correlation between correct rates across trials; for Experiment 1 only those subjects who completed both experiments were included (*N* = 19). **(B)** Correlation between Experiment 2 subject morph effects and correct rates across all trials. Morph effect calculated by subtracting correct rates over all minimum morphing distance trials from correct rates of all maximum distance trials.

Even though no timbre distance effect was found within attention conditions, the data displayed a trend toward the minimum timbre difference being more difficult than both maximum and intermediate timbre distances when segregating (bassoon and cello conditions), while the opposite was observed when integrating (aggregate condition). Further inspection of several contrasts testing for interaction effects did not indicate any differences (Supplementary Figure 3). However, when correlating subject morph effects, computed by subtracting maximum and minimum timbre distance scores, with their correct rates over all trials, those with lower overall scores showed the largest influence of morphing distance (*r* = 0.76, *p* < 0.001; Figure 5B), suggesting that behavioral effects of timbre distance may be masked in those subjects performing close to or at ceiling.

Comparing Experiment 2 LAB (0.90 [0.73 0.95], *Median [Inter Quartile Range]*) and SCAN (0.99 [0.94 1.0]) group data for correct rates over all trials, the addition of scanner noise to the scene did not have a detrimental effect on task performance; Experiment 1 LAB (0.90 [0.83 0.96]) and SCAN (0.92 [0.91 0.97]) group performance did not show differences.

## Discussion

We presented a novel paradigm developed to investigate both top-down and bottom-up modulations of auditory stream segregation and integration with custom-composed polyphonic music suitable for use by musically untrained listeners, and adaptable to neuroimaging protocols. In Experiment 1, subjects listened to two-part polyphonic music containing triplet patterns that served as attentional targets, and were instructed to attend to individual instruments (segregation), or to the aggregate (integration). Experiment 2 added a bottom-up modulation of instrument timbre distance into the attention-modulation framework of Experiment 1. Analysis of both Experiment 1 and Experiment 2 indicated that listeners were able to correctly identify the target in both tasks, after only modest training, at high performance levels. We observed no group-level performance difference between attentive conditions or instrument timbre distances, for both integration and segregation. In a subset of subjects, however, there appears to be a trend toward smaller timbre distances leading to a performance decrease, more specifically among those participants showing overall lower performance (Figure 5B), even though no significant interactions were found (Supplementary Figure 3). As demonstrated in Experiment 2, the addition of scanner noise to the task had no adverse effect on task performance, validating the task's suitability for fMRI studies with continuous pulse sequences. Aside from performance metrics, subjects tested in the scanner indicated having no difficulty segregating stimulus from scanner noise due to both the loudness difference and its continuous/repetitive nature. Overall, the findings demonstrated that non-musicians could be trained to both detect triplet patterns and reliably switch attention between scene elements, enabling the task to be employed in experiments studying stream segregation and integration in a natural listening context.

### Task considerations and future applications

High correct scores within our paradigm are desirable, both from the perspective of task compliance and task suitability for imaging experiments. We believe that a subject's capacity to detect the triplets correctly in individual voices, or across voices in the integrated condition, provides a strong indication they were either segregating or integrating, respectively. Each occurrence of triplets was incorporated into the melodic structure so as not to stand out from the surrounding music; this feature was explicitly designed during the composition in order to prevent any form of triplet pop-out, as this could lead to unintentional attention-target switches or alternative task strategies. The experimental design specifically focused on creating stimuli and task conditions which require listening effort, but are nonetheless feasible, to ensure that subjects are engaged in performing the desired task. Subjects reported that they needed strong attentional engagement to perform the task, both inside and outside of the scanner environment, especially due to their limited musical training. The subjects' capacity to detect the triplets correctly is primary evidence that they managed to segregate the mixture into individual streams. If listeners had not streamed the two melodies, they would not have been able to correctly respond whether the triplets were present or absent within a single instrumental voice. If the two streams are not segregated, the main remaining source of information differing between them would be rhythmic cues (tone onsets and offsets), based on which it would not be possible to assign the triplet's occurrence to one or the other of the voices. What we aim to investigate with this design is the brain's mechanisms which allows a listener to experience distinct melodic voices (or integrate across them) despite that the input arriving at the ear consists of a single mixed waveform of all sounds present in the scene.

Our paradigm was not designed for the detection of subject or condition differences, as demonstrated by a partial ceiling effect on the scores. Future iterations of the experimental protocol can possibly be sensitized to these effects by making the task more difficult for high-performing subjects. Pitch distance between melodic lines could, for example, be parametrically varied at an individual level to determine minimal pitch disparity needed for segregation (for examples see, van Noorden, 1977; Bregman, 1990) and hence maximal engagement of top-down processes. Conversely, pitch differences could be increased until segregation becomes almost inevitable for maximum reliance on bottom-up processes. Such designs would allow studying stream segregation and integration at various rates of top-down and bottom- up reliance. Increasing difficulty without adjustment of pitch could also be achieved by further reduction of timbre distance between instruments (for examples see, Bregman, 1990; Cusack and Roberts, 2000). As mentioned, timbre distance reduction appears to have a more pronounced effect on subjects who are not performing close to ceiling, compared to those who are (see Figures 3, 5B). This observation could be explained by a reduced cognitive load in high-performing subjects, allowing for compensation of the difficulty increase caused by timbre distance reduction, and possibly preventing emergence of a within-condition timbre effect and the hypothesized interaction effect. Insight into whether this hypothesis holds could be provided by testing these subjects with a timbre distance smaller than that based on their perceptual center points; alternatively, a higher tempo of the music could be adopted to make the task more challenging.

Due to the observed general performance increase/learning effect between Experiment 1 and Experiment 2 (Figure 5A), for future use we recommend that the two tasks be carried out in separate groups, possibly providing more challenging conditions and leading to a timbre-distance sensitivity of the performance metric. Subsequent task iterations could adopt the triplet presence or absence response immediately upon target detection, allowing, among others, the investigation of possible reaction time differences. Measuring reaction time could provide a handle on both intra- and inter-subject difficulty differences between conditions or trials, highlight possible cognitive load differences between subjects, and allow further investigation of whether specific stimuli are driving, or inhibiting, factors. A delayed response was adopted in the current setup to allow testing for task applicability to the neuroimaging setting, in which such a design is favored to reduce signal contamination by motor and decisional components. Even though the current sample showed better performance in the group tested in the scanner compared to the lab, we do not believe that subject performance is likely to be enhanced by being tested inside a large magnet, or by the presence of scanner noise. Most likely, observed group difference was due to sampling error; never the less this demonstrates that, at minimum, testing inside the fMRI scanner environment did not interfere with task performance. Presented experimental designs could provide a future vehicle to investigate plasticity and training effects in ASA with highly trained musicians. In order to perform experiments in an equally challenging ASA environment as for non-musicians, task design could be adjusted to include melodies synthesized by the same instrument which incorporate incorrect and/or incomplete cues in only one stream for the Aggregate condition, mis-tuned notes within triplets, or incomplete triplet patterns consisting of two or three triplet notes. Further differences may exist within musicians based on their specific training, with soloists possibly showing more difficulty with source segregation than conductors or orchestra members who are constantly separating their own instruments in the presence of multiple competing music streams, and a more general enhanced perceptual segregation of their main performing instrument (for example, Pantev et al., 2001; Carey et al., 2015). Further understanding of both subject's locus of attention and polyphonic listening behavior could be achieved by employing trials which contain a non-matching instruction and response, for example an instruction indicating attention to the aggregate and a response whether or not triplets were present in the bassoon.

Observed learning effects in subjects may be specific to the pitch ranges and instruments employed in the paradigm, not reflecting a general stream segregation performance gain which is transferable to other instruments or more common streaming tasks such as speech in noise detection. Within polyphonic music pieces, the upper voice is typically more salient (Palmer and Holleran, 1994; Crawley et al., 2002), suggesting that the segregation task may be more difficult when attending to the cello. Even though no performance difference is observed between the bassoon and cello condition in either of the experiments, verbal reports do confirm that subjectively listeners found the cello condition more difficult. In an attempt to control for the influence of timbre on the learning effects, timbres of voices could be switched to provide an indication whether learning was specific for the instrument-pitch relationship or resulted in general music streaming improvements. To try and reduce some specificity effects of learning, both the training and experimental melodies were uniquely written with a maximum variation in melody and pitch to maintain recognition as common polyphonic music while not destroying the similar pitch relation necessary amid voices across compositions. It is currently unknown whether performance on a music stream segregation task reflects general stream segregation or is more specific to music. A better understanding of their link could be achieved by performance comparison toward standardized complex sounds in background noise tasks such as speech in noise (for examples see, Kalikow et al., 1977; Nilsson et al., 1994; Wilson, 2003; Killion et al., 2004) or, in addition, a music-specific stream segregation measure such as the music in noise test (Coffey et al., 2017b). This would allow insight into whether music streaming employs similar mechanisms as the more widely established segregation task of isolating speech from background sounds. Subject performance will probably be very comparable on the music and speech in noise tasks, although an increase in subjects' musical training may cause larger performance gains on the music in noise paradigm compared to the speech in noise task.

### Polyphonic music perception

Several cognitive theories have been proposed to explain attention to polyphonic music, even though its neural processes are relatively unknown (for examples see, Janata et al., 2002; Ragert et al., 2014). Two of the main competing hypotheses are a divided attention (Gregory, 1990) and a figure-ground model (Sloboda and Edworthy, 2016). The divided attention model explains superior performance on polyphonic tasks, as compared to, for example, speech, by listeners' apparent capacity to divide their attentional resources over multiple melodic lines (Gregory, 1990). The figure-ground model, on the contrary, proposes that listeners attend only to a single melody while all others are assigned to the background (Sloboda and Edworthy, 2016), achieving multi-voiced perception by shifting their locus of attention between scene elements, therefore explaining perception via undivided attention. Contrary to what these models suggest, subjects are probably not simply dividing or alternating attention, they develop strategies to counteract divided attention issues by allowing for a true integration of melodies (Bigand et al., 2000). Even though listening strategies slightly differ between non-musicians, who appear to only integrate the melodic lines into streams, and musicians who are capable of constantly switching their attention between the integration and segregation of melodic lines, the general integrative model does appear to hold for both groups (Bigand et al., 2000), suggesting that attention to music may indeed differ from general auditory attention processes. The underlying neural attentional mechanism *per se* does probably not differ, it is the horizontal and vertical relationship which exists between melodies in combination with music-specific schema development which allows to both integrate and segregate music voices, further explaining musicians' superior performance on these tasks. Schema-based processes are developed on the basis of acquired knowledge and provide an additional form of top-down information important for stream formation (Bregman, 1990; Bey and McAdams, 2002), operating either in an attentive or pre-attentive mode, depending on task demands. Schemas have been shown to modulate music segregation performance in auditory scenes where integration is strongly driven by primitive (i.e., bottom-up) processes (Bregman, 1990; Bey and McAdams, 2002). When, for example, performing a segregation task with two interleaved melodies, it has been demonstrated that prior presentation of the to-be-attended sequence aids subsequent separation performance, while a frequency-transposition of the melody caused a reduction of these effects (Bey and McAdams, 2003). Within the current task we opted to not implement a modulation of top-down cues and focused on bottom-up attentive effects only, even though these cues are of great importance in ASA (McAdams and Bregman, 1979; Bregman, 1990; Micheyl et al., 2007; Ciocca, 2008) and could be employed to both investigate their contribution to music streaming and further aid or impede both the segregation and integration performance. Taken together, the interaction of both bottom-up and top-down processes appears to be capable of modulating whether subjects perceive multi-voiced music as integrated or segregated.

These considerations regarding musical stream segregation and the possible distinct mechanisms that are at play during music listening are also relevant for a broader understanding of how musical training may influence auditory cognition. For example, considerable evidence indicates that musicians outperform those without training in speech-in-noise tasks (Parbery-Clark et al., 2009; Swaminathan et al., 2015; Zendel et al., 2015, for review, see Coffey et al., 2017c). The neural mechanisms underlying this enhancement are not fully understood, even though there is evidence that both bottom-up mechanisms, centered within brainstem nuclei and auditory cortices (Bidelman et al., 2014; Coffey et al., 2017a), and top-down mechanisms (Kraus and Chandrasekaran, 2010), engaging motor and frontal-lobe systems (Du and Zatorre, 2017), play a role due to music's reliance on both kinds of processes. The task presented here could be used in conjunction with other tasks requiring segregation of targets from backgrounds to generate a better understanding of the relationship between music-specific auditory cognitive abilities, and their possible generalization to non-musical contexts.

## Conclusion

In this work we demonstrated that participants with limited to no musical education could be trained to isolate triplet patterns in polyphonic music, and complete both a selective attention task and a combined attention-timbre manipulation auditory streaming task with high accuracy. Triplet detection provided us with an objective variable assessing the listener's locus of attention, as well as their general task compliance, showing they were able to both successfully segregate individual instruments and integrate across the music streams. Insight into ASA processes with long complex music stimuli could be employed to inform research into, among others, hearing aid design and brain-based algorithm development for hearing aids, Brain Computer Interfaces, and provide a powerful means to investigate the neural mechanisms underlying both stream segregation and integration in naturalistic though well-controlled auditory scenes. An understanding of general ASA processes in the brain may very well be one of the necessary hurdles to cross in order to discern those processes underlying general music processing (Nelken, 2008).

## Ethics statement

This study was carried out with written informed consent from all subjects and in accordance with the recommendations of both the Montreal Neurological Institute and Hospital Research Ethics Board and the Maastricht University Ethics Review Committee Psychology and Neuroscience. All subjects gave written informed consent in accordance with the Declaration of Helsinki. The protocol was approved by the ethical committees of both universities.

## Author contributions

ND, RZ, EF, and GV designed the experiment; ND conducted the experiment; ND and GV analyzed the data; ND drafted the manuscript; ND, RZ, GV, and EF revised the manuscript.

### Conflict of interest statement

The authors declare that the research was conducted in the absence of any commercial or financial relationships that could be construed as a potential conflict of interest.

## Footnotes

^1^http://www.zlab.mcgill.ca

^2^http://www.wakayama-u.ac.jp/~kawahara/STRAIGHTadv/

^3^http://mcmc-jags.sourceforge.net/

^4^http://psiexp.ss.uci.edu/research/programs_data/jags/

## Supplementary material

Supplementary Figure 1 Mean pitch **(a)** and mean absolute frequency modulation **(b)** for bassoon (blue dots) and cello (green dots) per composition included in Experiment 1. Accuracy per composition across all trials for Experiment 1 **(c)** and Experiment 2 **(d)**; red horizontal line, median; box, 25th–75th percentile.

Supplementary Figure 2 Experiment 2 directed acyclic graph of Bayesian hierarchical model parameters for the d-prime model. Bias is modeled equally to d-prime, connecting to this model within the Hit and FA nodes. For simplicity, bias modeling is omitted from this graph. Notation based on Dietz (2010); see http://github.com/jluttine/tikz-bayesnet.

Supplementary Figure 3 Experiment 2 Bayesian hierarchical model, showing several possible contrasts and interactions; horizontal lines below histograms, 95% HDI.

Supplementary Figure 4 Experiment 2 condition specific Hit rates (squares) and False Alarm rates (dots) for group mean (colors) and individuals (black). Attention condition: Red, Aggregate; Blue, Bassoon; Green, Cello; Timbre distance: Max, maximum; Int, intermediate; Min, minimum.

## References

[B1] Alain C. Bernstein L. J. (2015). Auditory scene analysis: tales from cognitive neurosciences. Music Percept. 33, 70–82. 10.1525/mp.2015.33.1.70

[B2] Amaro E. Williams S. C. Shergill S. S. Fu C. H. Y. MacSweeney M. Picchioni M. M.. (2002). Acoustic noise and functional magnetic resonance imaging: current strategies and future prospects. J. Magn. Reson. Imaging 16, 497–510. 10.1002/jmri.10186 12412026

[B3] Andoh J. Ferreira M. Leppert I. R. Matsushita R. Pike B. Zatorre R. J. (2017). How restful is it with all that noise? Comparison of Interleaved silent steady state (ISSS) and conventional imaging in resting-state fMRI. Neuroimage 147, 726–735. 10.1016/j.neuroimage.2016.11.065 27902936

[B4] Belin P. Zatorre R. J. Hoge R. Evans A. C. Pike B. (1999). Event-related fMRI of the auditory cortex. Neuroimage 10, 417–429. 10.1006/nimg.1999.0480 10493900

[B5] Besle J. Schevon C. A. Mehta A. D. Lakatos P. Goodman R. R. McKhann G. M.. (2011). Tuning of the human neocortex to the temporal dynamics of attended events. J. Neurosci. 31, 3176–3185. 10.1523/JNEUROSCI.4518-10.2011 21368029 PMC3081726

[B6] Bey C. McAdams S. (2002). Schema-based processing in auditory scene analysis. Percept. Psychophys. 64, 844–854. 10.3758/BF03194750 12201342

[B7] Bey C. McAdams S. (2003). Postrecognition of interleaved melodies as an indirect measure of auditory stream formation. J. Exp. Psychol. Hum. Percept. Perform. 29, 267–279. 10.1037/0096-1523.29.2.267 12760614

[B8] Bidelman G. M. Weiss M. W. Moreno S. Alain C. (2014). Coordinated plasticity in brainstem and auditory cortex contributes to enhanced categorical speech perception in musicians. Eur. J. Neurosci. 40, 2662–2673. 10.1111/ejn.12627 24890664

[B9] Bigand E. Foret S. McAdams S. (2000). Divided attention in music. Int. J. Psychol. 35, 270–278. 10.1080/002075900750047987

[B10] Bregman A. S. (1990). Auditory Scene Analysis, The Perceptual Organization of Sound. Cambridge, MA: MIT Press.

[B11] Bregman A. S. (2015). Progress in understanding auditory scene analysis. Music Percept. 33, 12–19. 10.1525/mp.2015.33.1.12

[B12] Bregman A. S. Pinker S. (1978). Auditory streaming and the building of timbre. Can. J. Psychol. 32, 19–31. 728845 10.1037/h0081664

[B13] Brochard R. Drake C. Botte M. C. McAdams S. (1999). Perceptual organization of complex auditory sequences: effect of number of simultaneous subsequences and frequency separation. J. Exp. Psychol. Hum. Percept. Perform. 25, 1742–1759. 10.1037/0096-1523.25.6.1742 10641316

[B14] Carey D. Rosen S. Krishnan S. Pearce M. T. Shepherd A. Aydelott J.. (2015). Generality and specificity in the effects of musical expertise on perception and cognition. Cognition 137, 81–105. 10.1016/j.cognition.2014.12.005 25618010

[B15] Carlyon R. P. (2003). How the brain separates sounds. Trends Cogn. Sci. 8, 465–471. 10.1016/j.tics.2004.08.008 15450511

[B16] Carlyon R. P. Cusack R. (2005). Effects of attention on auditory perceptual organization, in Neurobiology of Attention, eds Itti L. Rees G. Tsotos J. K. (Cambrige, MA: Elsevier), 317–323.

[B17] Ciocca V. (2008). The auditory organization of complex sounds. Front. Biosci. 13, 148–169. 10.2741/2666 17981534

[B18] Coffey E. B. J. Chepesiuk A. M. P. Herholz S. C. Baillet S. Zatorre R. J. (2017a). Neural correlates of early sound encoding and their relationship to speech-in-noise perception. Front. Neurosci. 11:479. 10.3389/fnins.2017.00479 28890684 PMC5575455

[B19] Coffey E. B. J. Lim A. R. Zatorre R. J. (2017b). The music-in-noise task: a tool for dissecting complex auditory perception, in The Neurosciences and Music VI Music, Sound, and Health (Boston, MA), 5–18.

[B20] Coffey E. B. J. Mogilever N. B. Zatorre R. J. (2017c). Speech-in-noise perception in musicians: a review. Hear. Res. 352, 49–69. 10.1016/j.heares.2017.02.006 28213134

[B21] Coffey E. B. J. Scala S. Zatorre R. J. (2011). Montreal Music History Questionnaire: a tool for the assessment of music-related experience, in Neurosciences and Music IV Learning and Memory (Edinburgh), 9–12.

[B22] Crawley E. J. Acker-Mills B. E. Pastore R. E. Weil S. (2002). Change detection in multi-voice music: the role of musical structure, musical training, and task demands. J. Exp. Psychol. Hum. Percept. Perform. 28, 367–378. 10.1037/0096-1523.28.2.367 11999860

[B23] Cusack R. Decks J. Aikman G. Carlyon R. P. (2004). Effects of location, frequency region, and time course of selective attention on auditory scene analysis. J. Exp. Psychol. Hum. Percept. Perform. 30, 643–656. 10.1037/0096-1523.30.4.643 15301615

[B24] Cusack R. Roberts B. (2000). Effects of differences in timbre on sequential grouping. Percept. Psychophys. 62, 1112–1120. 10.3758/BF03212092 10997053

[B25] Deutsch D. (2010). Hearing music in ensembles. Phys. Today 63, 40–45. 10.1063/1.3326988

[B26] Deutsch D. (2013). Grouping mechanisms in music, in The Psychology of Music, ed Deutsch D. (London: Elsevier), 183–248.

[B27] Dietz L. (2010). Directed factor graph notation for generative models. Max Planck Institute for Informatics, Technical Report.

[B28] Du Y. Zatorre R. J. (2017). Musical training sharpens and bonds ears and tongue to hear speech better. Proc. Natl. Acad. Sci. U.S.A. 114, 13579–13584. 10.1073/pnas.1712223114 29203648 PMC5754781

[B29] Gregory A. H. (1990). Listening to polyphonic music. Psychol. Music 18, 163–170. 10.1177/0305735690182005

[B30] Gregory A. H. (1994). Timbre and auditory streaming. Music Percept. 12, 161–174. 10.2307/40285649

[B31] Hall A. J. Brown T. A. Grahn J. A. Gati J. S. Nixon P. L. Hughes S. M. (2014). There's more than one way to scan a cat: imaging cat auditory cortex with high-field fMRI using continuous or sparse sampling. J. Neurosci. Methods 224, 96–106. 10.1016/j.jneumeth.2013.12.012 24389047

[B32] Janata P. Tillmann B. Bharucha J. J. (2002). Listening to polyphonic music recruits domain-general attention and working memory circuits. Cogn. Affect. Behav. Neurosci. 2, 121–140. 10.3758/CABN.2.2.121 12455680

[B33] Kalikow D. N. Stevens K. N. Elliott L. L. (1977). Development of a test of speech intelligibility in noise using sentence materials with controlled word predictability. J. Acoust. Soc. Am. 61, 1337–1351. 10.1121/1.381436 881487

[B34] Kawahara H. Matsui H. (2003). Auditory morphing based on an elastic perceptual distance metric in an interference-free time-frequency representation, in International Conference on Acoustics, Speech, and Signal Processing, 2003 (Hong Kong: IEEE), 256–259.

[B35] Killion M. C. Niquette P. A. Gudmundsen G. I. Revit L. J. Banerjee S. (2004). Development of a quick speech-in-noise test for measuring signal-to-noise ratio loss in normal-hearing and hearing-impaired listeners. J. Acoust. Soc. Am. 116, 2395–2405. 10.1121/1.1784440 15532670

[B36] Kraus N. Chandrasekaran B. (2010). Music training for the development of auditory skills. Nat. Rev. Neurosci. 11, 599–605. 10.1038/nrn2882 20648064

[B37] Kruschke J. K. (2014). Doing Bayesian Data Analysis, A Tutorial with R, JAGS, and Stan, 2 Edn. London: Academic Press.

[B38] Lakatos P. Musacchia G. O'Connel M. N. Falchier A. Y. Javitt D. C. Schroeder C. E. (2013). The spectrotemporal filter mechanism of auditory selective attention. Neuron 77, 750–761. 10.1016/j.neuron.2012.11.034 23439126 PMC3583016

[B39] Marozeau J. Innes-Brown H. Blamey P. J. (2013). The effect of timbre and loudness on melody segregation. Music Percept. 30, 259–274. 10.1525/mp.2012.30.3.259

[B40] McAdams S. (2013a). Musical timbre perception, in The Psychology of Music, ed Deutsch D. (London: Elsevier Inc.), 35–68.

[B41] McAdams S. (2013b). Timbre as a structuring force in music, in ICA 2013 Montreal (Montreal, QC: ASA), 1–6.

[B42] McAdams S. Bregman A. S. (1979). Hearing musical streams. Comput. Music J. 3, 26–43.

[B43] McDermott J. H. Oxenham A. J. (2008). Music perception, pitch, and the auditory system. Curr. Opin. Neurobiol. 18, 452–463. 10.1016/j.conb.2008.09.005 18824100 PMC2629434

[B44] Melara R. D. Marks L. E. (1990). Interaction among auditory dimensions: timbre, pitch, and loudness. Percept. Psychophys. 48, 169–178. 10.3758/BF03207084 2385491

[B45] Micheyl C. Carlyon R. P. Gutschalk A. Melcher J. R. Oxenham A. J. Rauschecker J. P.. (2007). The role of auditory cortex in the formation of auditory streams. Hear. Res. 229, 116–131. 10.1016/j.heares.2007.01.007 17307315 PMC2040076

[B46] Moore B. C. J. Gockel H. (2002). Factors influencing sequential stream segregation. Acta Acust. Unit. Acust. 88, 320–333. Available online at: http://www.ingentaconnect.com/content/dav/aaua/2002/00000088/00000003/art00004

[B47] Nelken I. (2008). Neurons and objects: the case of auditory cortex. Front. Neurosci. 2:107. 10.3389/neuro.01.009.2008 18982113 PMC2570071

[B48] Nilsson M. Soli S. D. Sullivan J. A. (1994). Development of the Hearing In Noise Test for the measurement of speech reception thresholds in quiet and in noise. J. Acoust. Soc. Am. 95, 1085–1099. 10.1121/1.408469 8132902

[B49] Palmer C. Holleran S. (1994). Harmonic, melodic, and frequency height influences in the perception of multivoiced music. Percept. Psychophys. 56, 301–312. 10.3758/BF03209764 7971130

[B50] Pantev C. Roberts L. E. Schulz M. Engelien A. (2001). Timbre-specific enhancement of auditory cortical representations in musicians. Neuroreport 12, 169–174. 10.1097/00001756-200101220-00041 11201080

[B51] Parbery-Clark A. Skoe E. Lam C. Kraus N. (2009). Musician enhancement for speech-in-noise. Ear Hear. 30, 653–661. 10.1097/AUD.0b013e3181b412e9 19734788

[B52] Peretz I. Zatorre R. J. (2005). Brain organization for music processing. Annu. Rev. Psychol. 56, 89–114. 10.1146/annurev.psych.56.091103.070225 15709930

[B53] Ragert M. Fairhurst M. T. Keller P. E. (2014). Segregation and integration of auditory streams when listening to multi-part music. PLoS ONE 9:e84085. 10.1371/journal.pone.0084085 24475030 PMC3901649

[B54] Riecke L. Peters J. C. Valente G. Kemper V. G. Formisano E. Sorger B. (2016). Frequency-selective attention in auditory scenes recruits frequency representations throughout human superior temporal cortex. Cereb. Cortex 27, 3002–3014. 10.1093/cercor/bhw160 27230215

[B55] Shamma S. A. Micheyl C. (2010). Behind the scenes of auditory perception. Curr. Opin. Neurobiol. 20, 361–366. 10.1016/j.conb.2010.03.009 20456940 PMC2901988

[B56] Sloboda J. Edworthy J. (2016). Attending to two melodies at once: the of key relatedness. Psychol. Music 9, 39–43. 10.1177/03057356810090010701

[B57] Stanislaw H. Todorov N. (1999). Calculation of signal detection theory measures. Behav. Res. Methods Instrum. Comput. 31, 137–149. 10.3758/BF03207704 10495845

[B58] Sussman E. S. (2005). Integration and segregation in auditory scene analysis. J Acoust. Soci. Am. 117, 1285–14. 10.1121/1.1854312 15807017

[B59] Sussman E. S. Horváth J. Winkler I. Orr M. (2007). The role of attention in the formation of auditory streams. Percept. Psychophys. 69, 136–152. 10.3758/BF03194460 17515223

[B60] Swaminathan J. Mason C. R. Streeter T. M. Best V. Gerald Kidd J. Patel A. D. (2015). Musical training, individual differences and the cocktail party problem. Sci. Rep. 5:14401 10.1038/srep14401 26112910 PMC4481518

[B61] Uhlig M. Fairhurst M. T. Keller P. E. (2013). The importance of integration and top-down salience when listening to complex multi-part musical stimuli. Neuroimage 77, 52–61. 10.1016/j.neuroimage.2013.03.051 23558103

[B62] van Noorden L. P. A. S. (1977). Minimum differences of level and frequency for perceptual fission of tone sequences ABAB. J. Acoust. Soc. Am. 61, 1041–1045. 10.1121/1.381388 864091

[B63] Wessel D. L. (1979). Timbre space as a musical control structure. Comput. Music J. 3, 45–52. 10.2307/3680283

[B64] Wilson R. H. (2003). Development of a speech-in-multitalker-babble paradigm to assess word-recognition performance. J. Am. Acad. Audiol. 14, 453–470. Available online at: http://www.ingentaconnect.com/content/aaa/jaaa/2003/00000014/00000009/art00002 14708835

[B65] Zatorre R. J. Zarate J. M. (2012). Cortical processing of music, in The Human Auditory Cortex (New York, NY: Springer), 261–294.

[B66] Zendel B. R. Tremblay C.-D. Belleville S. Peretz I. (2015). The impact of musicianship on the cortical mechanisms related to separating speech from background noise. J. Cogn. Neurosci. 27, 1044–1059. 10.1162/jocn_a_00758 25390195

