# Supplementary figures and images for: Assessing Top-Down and Bottom-Up Contributions to Auditory Stream Segregation and Integration With Polyphonic Music
Source: Front Neurosci. 2018 Mar 7;12:121. doi: 10.3389/fnins.2018.00121 (PMC5845899; doi:10.3389/fnins.2018.00121)

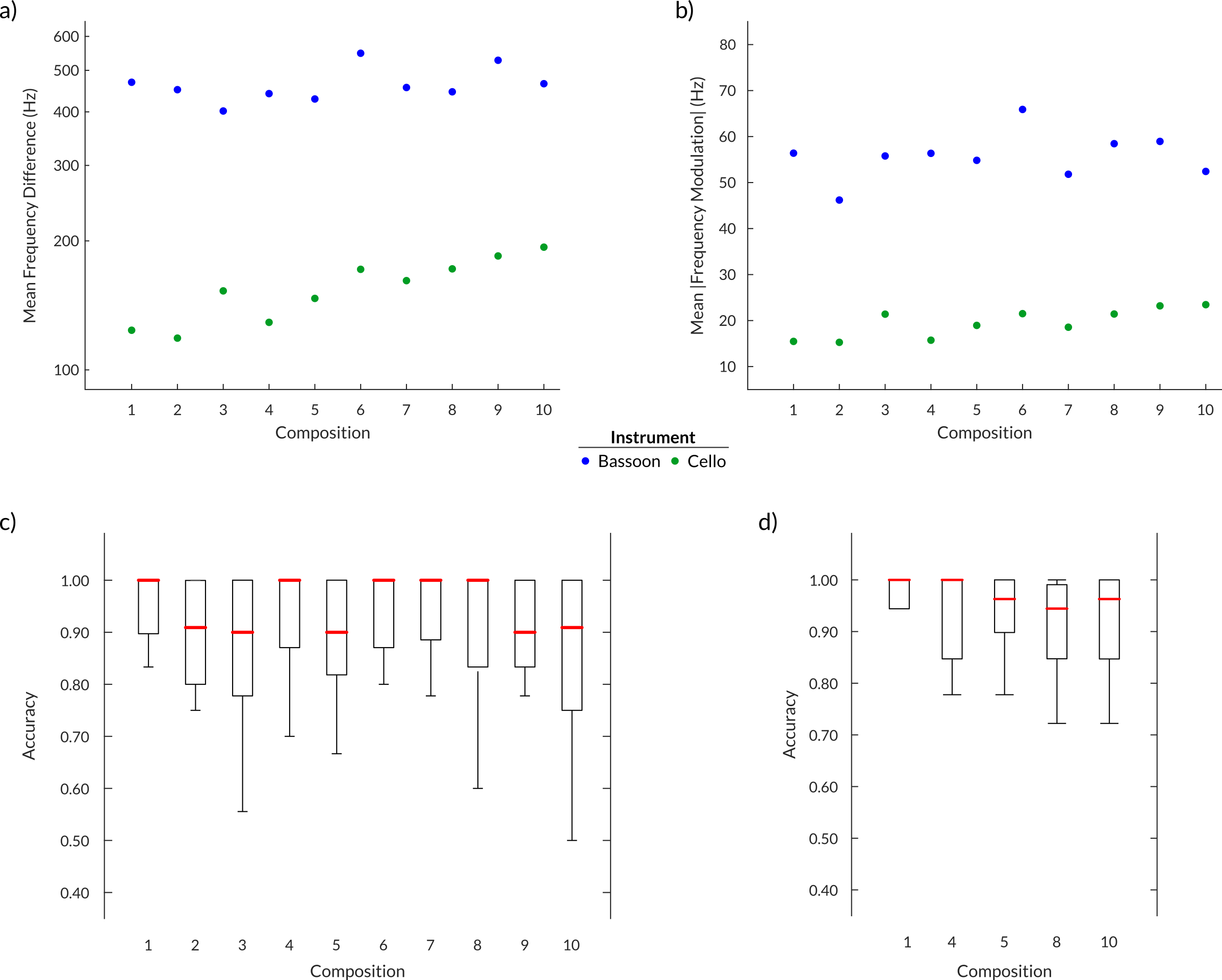

Supplement: Supplementary Figure 1 — Mean pitch (a) and mean absolute frequency modulation (b) for bassoon (blue dots) and cello (green dots) per composition included in Experiment 1. Accuracy per composition across all trials for Experiment 1 (c) and Experiment 2 (d); red horizontal line, median; box, 25th–75th percentile. [file Image1.tiff]

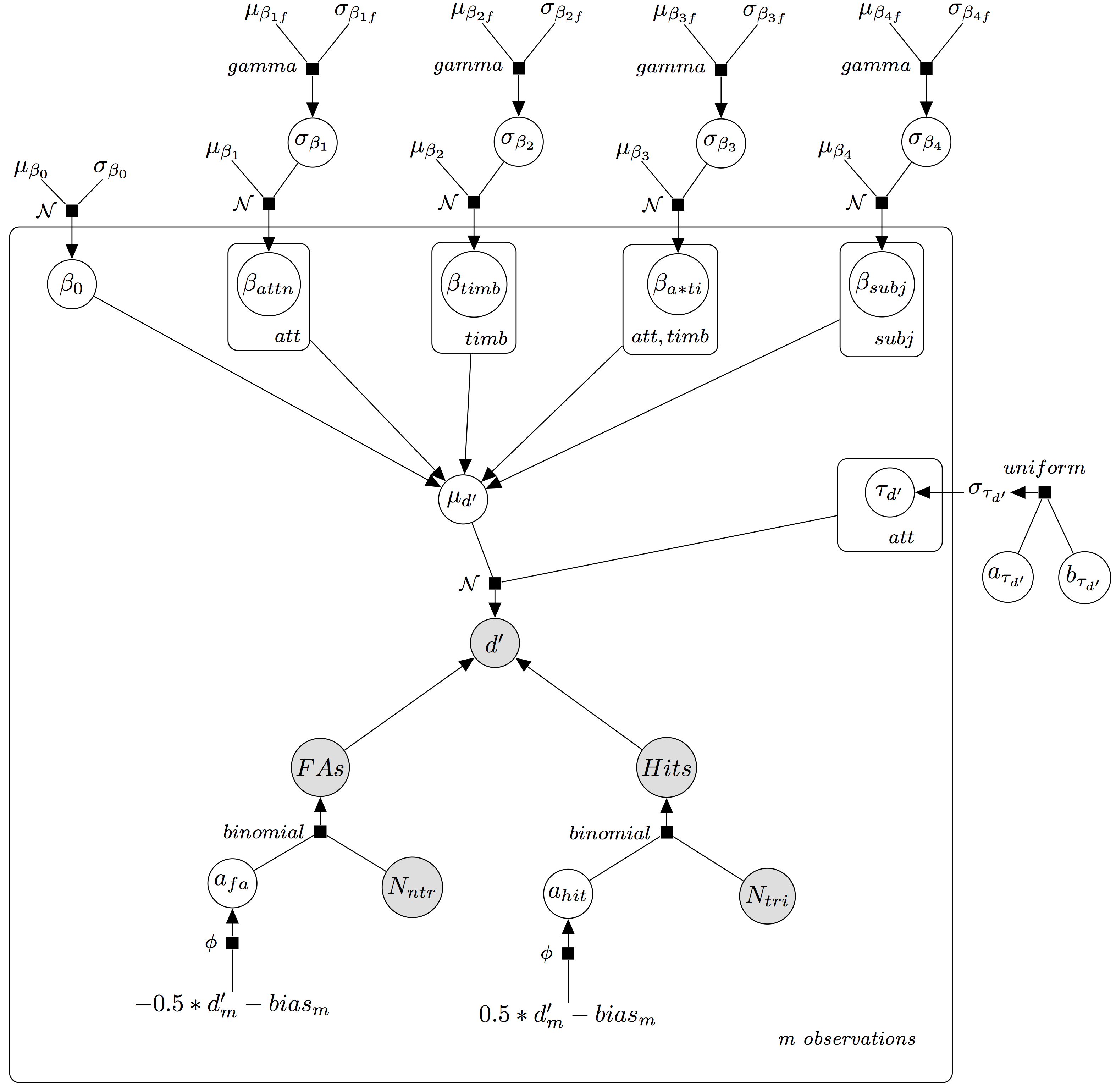

Supplement: Supplementary Figure 2 — Experiment 2 directed acyclic graph of Bayesian hierarchical model parameters for the d-prime model. Bias is modeled equally to d-prime, connecting to this model within the Hit and FA nodes. For simplicity, bias modeling is omitted from this graph. Notation based on Dietz (2010); see http://github.com/jluttine/tikz-bayesnet. [file Image2.tiff]

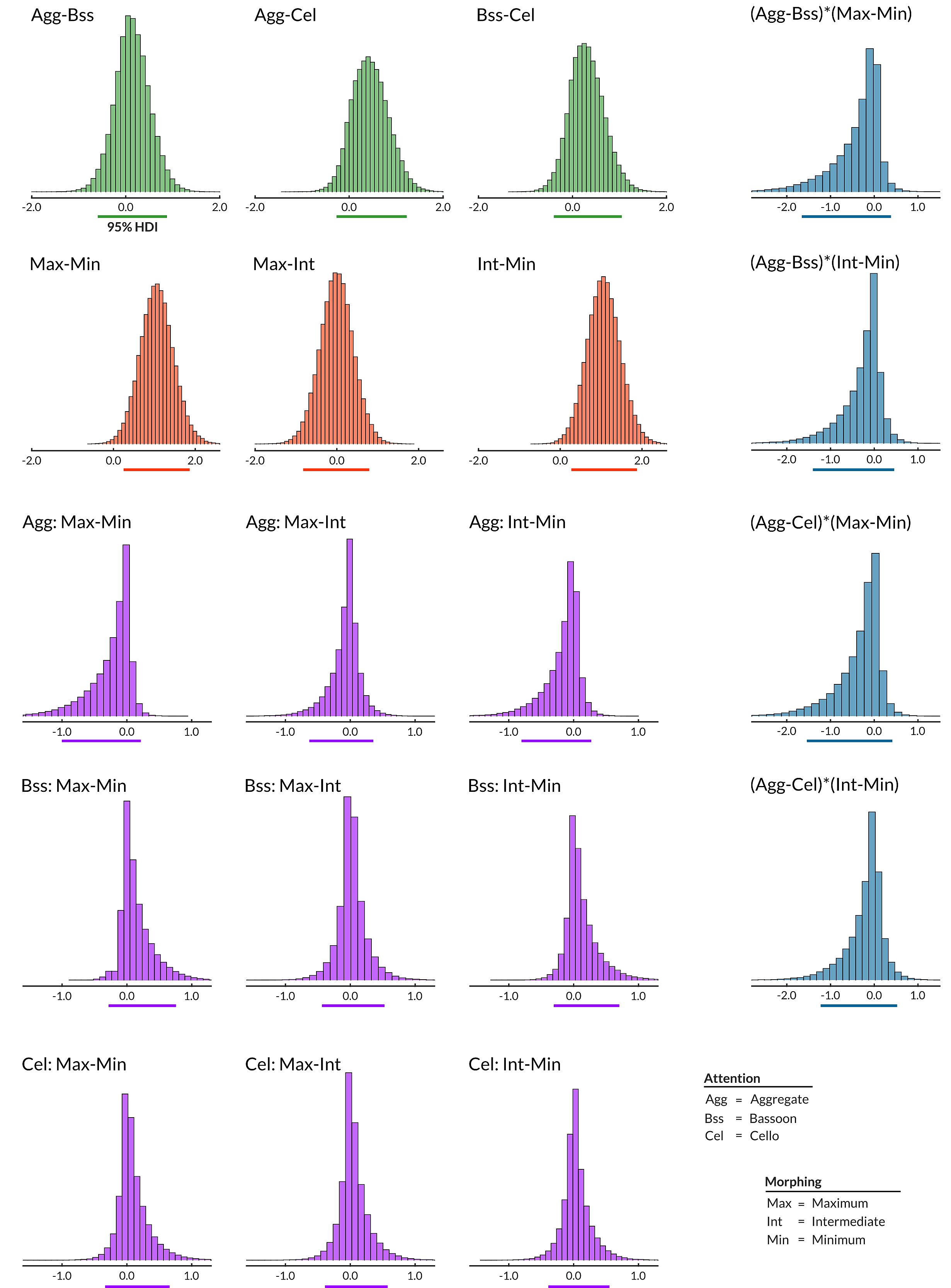

Supplement: Supplementary Figure 3 — Experiment 2 Bayesian hierarchical model, showing several possible contrasts and interactions; horizontal lines below histograms, 95% HDI. [file Image3.tiff]

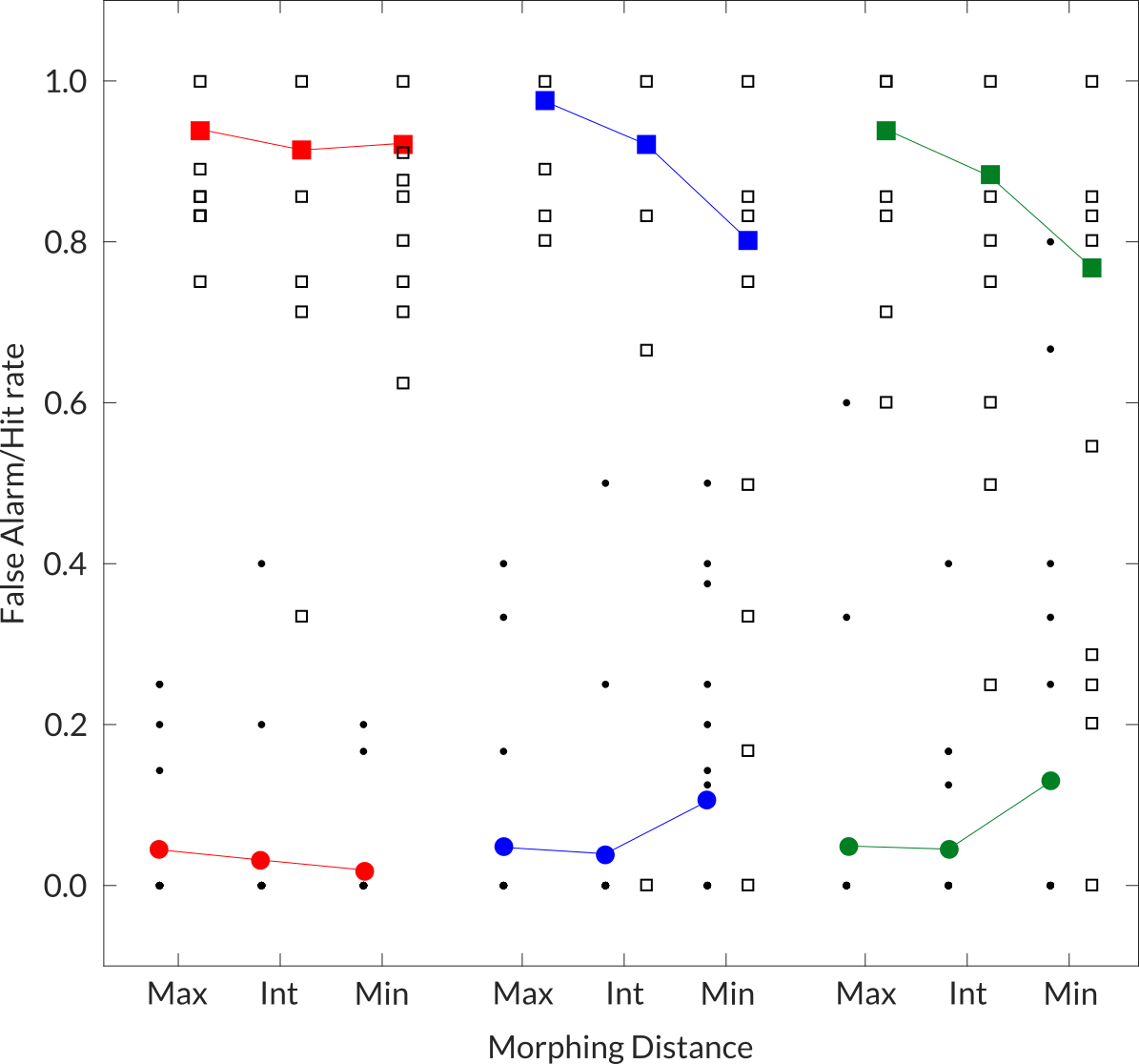

Supplement: Supplementary Figure 4 — Experiment 2 condition specific Hit rates (squares) and False Alarm rates (dots) for group mean (colors) and individuals (black). Attention condition: Red, Aggregate; Blue, Bassoon; Green, Cello; Timbre distance: Max, maximum; Int, intermediate; Min, minimum. [file Image4.tiff]
